# Depression and Anxiety Symptoms Among Cisgender Gay and Bisexual Men During the Onset of the COVID-19 Pandemic: Time Series Analysis of a US National Cohort Study

**DOI:** 10.2196/47048

**Published:** 2024-01-26

**Authors:** Chloe Mirzayi, Drew Westmoreland, Matthew Stief, Christian Grov

**Affiliations:** 1 CUNY Graduate School of Public Health and Health Policy New York, NY United States

**Keywords:** COVID-19 pandemic, lesbian, gay, bisexual, transgender, and queer, LGBTQ, mental health, depression, anxiety, time series, gay and bisexual men, cisgender gay, pandemic, gay

## Abstract

**Background:**

The onset of the COVID-19 pandemic in the United States in March 2020 caused a dramatic change in the way many people lived. Few aspects of daily life were left undisrupted by the pandemic’s onset as well as the accompanying policies to control the spread of the disease. Previous research has found that the pandemic may have significantly impacted the mental health of lesbian, gay, bisexual, transgender, and queer (LGBTQ) individuals—potentially more so than other individuals. However, the pandemic did not affect all areas of the United States at the same time, and there may be regional variation in the impact of the onset of the pandemic on depressive symptoms among LGBTQ individuals.

**Objective:**

To assess regional variation of the impact of the pandemic, we conducted a time series analysis stratified by US geographic region to examine symptoms of depression and anxiety among a sample of primarily cisgender gay and bisexual men before and after the onset of the COVID-19 pandemic in the United States.

**Methods:**

In total, 5007 participants completed assessments as part of the *Together 5000* study, an ongoing prospective cohort study. Depressive and anxiety symptoms were measured using the Patient Health Questionnaire-4. Patient Health Questionnaire-4 scores were graphed as a function of days from March 15, 2020. Locally estimated scatterplot smoothing trend lines were applied. A sieve-bootstrap Mann-Kendall test for monotonic trend was conducted to assess the presence and direction of trends in the scatterplots. We then compared the observed trends to those observed for 1 year prior (2018-2019) to the pandemic onset using data collected from the same sample.

**Results:**

Significant positive trends were detected for the Northeast (*P*=.03) and Midwest (*P*=.01) regions of the United States in the 2020 assessment, indicating that symptoms of anxiety and depression were increasing in the sample in these regions immediately prior to and during the onset of the pandemic. In contrast, these trends were not present in data from the 2018 to 2019 assessment window.

**Conclusions:**

Symptoms of anxiety and depression increased among the study population in the Northeast and Midwest during the beginning months of the COVID-19 pandemic, but similar increase was not observed in the South and West regions. These trends were also not found for any region in the 2018 to 2019 assessment window. This may indicate region-specific trends in anxiety and depression, potentially driven by the burden of the pandemic and policies that varied from region to region. Future studies should consider geographic variation in COVID-19 spread and policies as well as explore potential mechanisms by which this could influence the mental health of LGBTQ individuals.

## Introduction

The onset of the COVID-19 pandemic in the United States in early 2020 represented a nearly unprecedented disruption in daily life for many Americans. Widespread disruptions were present in nearly every facet of daily life, including in workplaces, schools, and social settings. Mandatory business closures and recommended social distancing were implemented with some heterogeneity in the United States. Many states in the Northeast, Midwest, and West implemented stay-at-home orders in March 2020 prior to Southern states, where the pandemic later took hold in the summer [1].

Several large studies have linked the onset of the pandemic to a significant increase in the symptoms of depression and anxiety in US adults [2-5]. Other studies have found that lesbian, gay, bisexual, transgender, and queer (LGBTQ) people may be particularly vulnerable to developing symptoms of depression and anxiety relative to non-LGBTQ populations [6]. For instance, a study of cannabis users conducted during the pandemic found that the LGBTQ cannabis users were more likely to report symptoms of depression and anxiety [7]. Similarly, a national longitudinal cohort study of LGTBQ adults found increased anxiety and depression symptoms among participants after the beginning of the pandemic [8]. Additionally, a study of LGBTQ college students found that nearly half had unmet mental health needs during the first few months of the pandemic [6]. These findings may be due to being confined in uncomfortable home situations or isolation from friends, which may have disparate impacts on LGBTQ individuals compared to others [9]. However, other studies have found decreased anxiety symptoms and no differences in depressive symptoms before and after the start of the pandemic [10].

Recently, researchers have proposed potential mechanisms by which the onset of the COVID-19 pandemic may have disproportionately affected LGBTQ people. A web-based study in Germany during the pandemic found that depressive symptoms were mediated by loneliness [11], while a study of Mexican LGBTQ people found that perceived social support also acted as a mediator [12]. Taken together, it appears that a lack of access to supportive friends and other members of the LGBTQ community, and the accompanying loneliness, may be one mechanism through which the COVID-19 pandemic, and associated lockdowns, had a greater impact on LGBTQ individuals compared to others.

Despite several studies finding that LGBTQ people were more likely to experience symptoms of depression and anxiety with the onset of the COVID-19 pandemic, we have been unable to identify any studies that examined trends in symptoms and anxiety over time for the period. Time series studies have been used to examine trends in mental health leading up to and immediately following the 2016 US presidential election [13], in mental health referrals during the pandemic [14,15], and in suicide mortality also during the COVID-19 pandemic [16]. However, to our knowledge, no study has examined symptoms of depression and anxiety in an LGBTQ population during the onset of the COVID-19 pandemic in the United States using a time series approach.

The aim of this study was to examine the effects of the onset of the first wave of the COVID-19 pandemic on a population of primarily gay and bisexual men, but also transgender men and transgender women as well as nonbinary people, using time series analysis. Given the heterogeneity of the burden of cases and in COVID-19 policies, analyses were stratified by US census region. We hypothesized that regions that were more impacted by the early onset of the COVID-19 pandemic or had more stringent pandemic control policies or recommendations (eg, the Northeast) would show an increase in symptoms of depression and anxiety as the pandemic progressed.

## Methods

### Study Population

Participants were members of *Together 5000*, a US national cohort of primarily cisgender gay and bisexual men at risk for HIV recruited from sexual networking applications. Members of the cohort complete yearly web-based surveys as well as at-home HIV testing beginning in 2017 and running through the onset of the pandemic. The yearly web-based surveys assessed a variety of health-related domains such as socioeconomic factors, drug and alcohol use, sexual behaviors, food and housing insecurity, and mental health measures. A detailed profile of the study procedures and participants has been published elsewhere [17].

### Measures

#### Data Collection

Data for this study were primarily taken from participants’ 24-month assessment. These assessments began on November 20, 2019 (–116 days), and ran through August 15, 2020 (+153 days)—with some participants completing their assessment prior to the onset of the COVID-19 pandemic in the United States and others completing it after. We defined the onset as March 15, 2020, which roughly coincides with the beginning of the week in which many local and state governments announced mandatory closure of nonessential businesses and schools [18-20].

To establish whether a similar trend would be observed in prior yearly assessments, we compared 24-month results to participants’ 12-month assessment (all of which were conducted prior to the pandemic). Survey completion dates were calculated relative to March 15, 2019, and ranged from November 7, 2018 (–127 days), to June 24, 2019 (+101 days).

#### Demographics

On the baseline survey, race and ethnicity information was assessed using a list of common racial and ethnic categories for participants to select all that apply. Participants were also given the ability to specify their own race or ethnicity. These options were recoded to a 4-category variable representing Black, Hispanic, White, and multiracial or others. To assess current gender identity, participants were asked their assigned sex at birth as well as their current gender identity. Participants had the option of writing in their own gender identity. The variable was then recoded into 5 categories: cisgender man, cisgender woman, transgender man, transgender woman, or others or nonbinary. Finally, participants were asked to select the best option for their sexual orientation: gay, queer, or homosexual; bisexual; straight or heterosexual; or others. This variable was not recoded prior to analysis.

#### Region

Participants self-reported their zip codes at baseline and were able to provide updated zip codes during the 12- and 24-month assessments. These zip codes were coded into state of residence, which in turn were categorized according to US census regions [21].

#### Patient Health Questionnaire-4

The 4-item Likert-type Patient Health Questionnaire-4 (PHQ-4) was used to assess symptoms of anxiety and depression. The Patient Health Questionnaire-2, a measure of depression, and Generalized Anxiety Disorder-2, a measure of anxiety, constitute the PHQ-4. The reliability and validity of the PHQ-4 in the general population have been previously established [22]. The PHQ-4 is scored from 0 to 12, with an increasing score indicating increasingly severe anxiety and depressive symptomology. The continuous measure was used for analyses.

### Statistical Analysis

PHQ-4 scores were stratified by US census region of residence and then plotted as a function of the survey completion date for participants completing the 24-month assessment, which ran from November 20, 2019, to August 15, 2020. A locally estimated scatterplot smoothing trend line with 95% CI was applied. A sieve-bootstrap Mann-Kendall test for monotonic trends was conducted to assess the presence and direction of trends in the scores. All steps of this analysis were then repeated for the 12-month assessment for comparison.

Analyses were conducted in R (version 4.1.0; R Foundation for Statistical Computing). Plots and trend lines were created using the *ggplot2* package [23], while the test for trend was conducted using the *funtimes* package [24].

### Ethical Considerations

All procedures performed in studies involving human participants were in accordance with the ethical standards of the institutional review board (Institutional Review Board of the City University of New York Graduate School of Public Health and Health Policy, IRB file #2017-0893) and with the 1964 Helsinki declaration and its later amendments or comparable ethical standards. All participants provided informed consent for data collection at each assessment. Participant data were deidentified and stored in an encrypted closed network. Participants received US $25 Amazon gift cards for completing study assessments.

## Results

In total, 5007 participants completed the 24-month assessment survey and resided in 1 of the 4 US census regions. An additional 22 participants completed the survey but resided in a US territory and were not included in these analyses. Of these 5007 participants, 784 (15.6%) resided in the Northeast, 780 (15.6%) resided in the Midwest, 2285 (45.6%) resided in the South, and 1158 (23.1%) resided in the West.

Demographically, 507 (10.1%) participants were Black, 1213 (24.2%) were Hispanic, 2677 (53.5%) were White, and 610 (12.2%) were another race or multiracial. The mean age of the sample at the 24-month assessment was 32.9 (SD 7.5) years. The sample was primarily cisgender men with 4885 (97.6%) identifying as such, with the remaining 122 (2.4%) participants identifying as transgender men, transgender women, or nonbinary. A substantial proportion of the sample identified as gay, queer, or homosexual (n=4291, 85.7%) or bisexual (n=660, 13.2%), with the remainder identifying as others. Full demographics as well as PHQ-4 scores stratified by demographic variables are presented in Table 1.

The mean PHQ-4 score at the 24-month assessment was 4.0 (SD 3.0), while the mean PHQ-4 score at the 12-month assessment was 3.7 (SD 2.4). A paired 2-tailed *t* test revealed this difference to be statistically significant (*t*_4653_=2.99; *P*=.019), indicating an overall increase in PHQ-4 scores between the 12-month assessment and the 24-month assessment.

Survey completion dates for the 24-month assessment were calculated relative to the onset of the COVID-19 pandemic, with a 0 representing March 15, 2020. These dates ranged from November 20, 2019, to August 15, 2020. Individual participant Generalized Anxiety Disorder–Patient Health Questionnaire scores were then plotted as a function of survey completion dates stratified by US census region.

**Figure 1:**
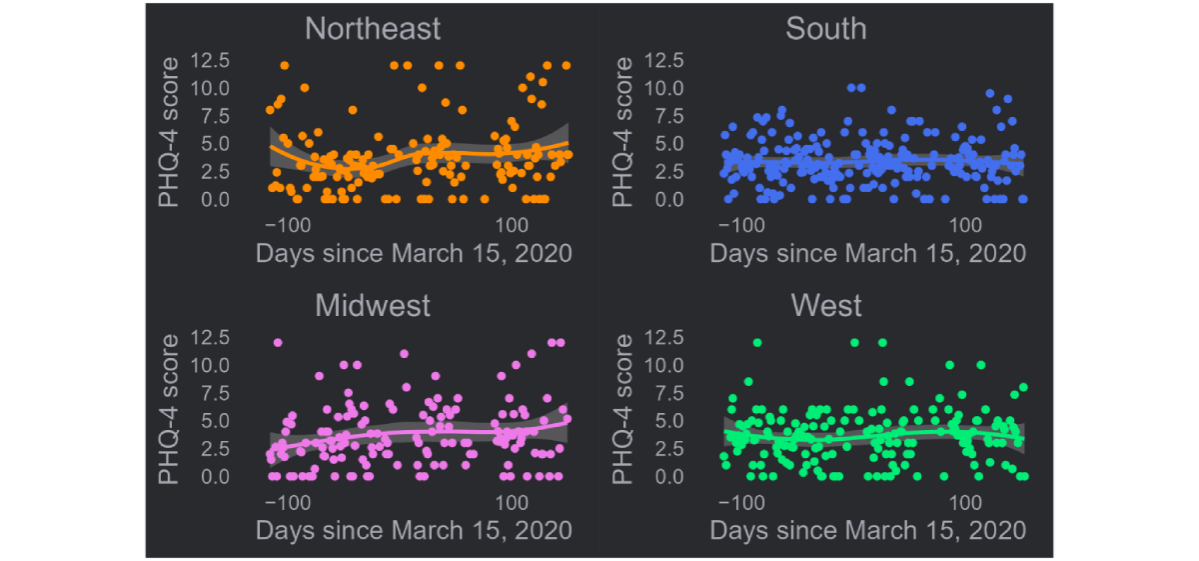
US census region–stratified trends in PHQ-4 scores over time before and after the onset of the COVID-19 pandemic of a prospective US national cohort study of primarily cisgender gay and bisexual men in 2020. PHQ-4: Patient Health Questionnaire-4.

The locally estimated scatterplot smoothing trend line in PHQ-4 scores for the Northeast revealed a trend downward in the days immediately prior to the onset of the pandemic with an upturn in scores following the onset (Figure 1). This trend was significant (τ=0.12; *P*=.03). A small, steady increase in scores was also observed among participants in the Midwest, beginning prior to the pandemic onset and continuing through to the summer (τ=0.14; *P*=.01). No trends are immediately apparent in the trend lines for the South and West, and tests for trends were not statistically significant (Table 2).

**Figure 2:**
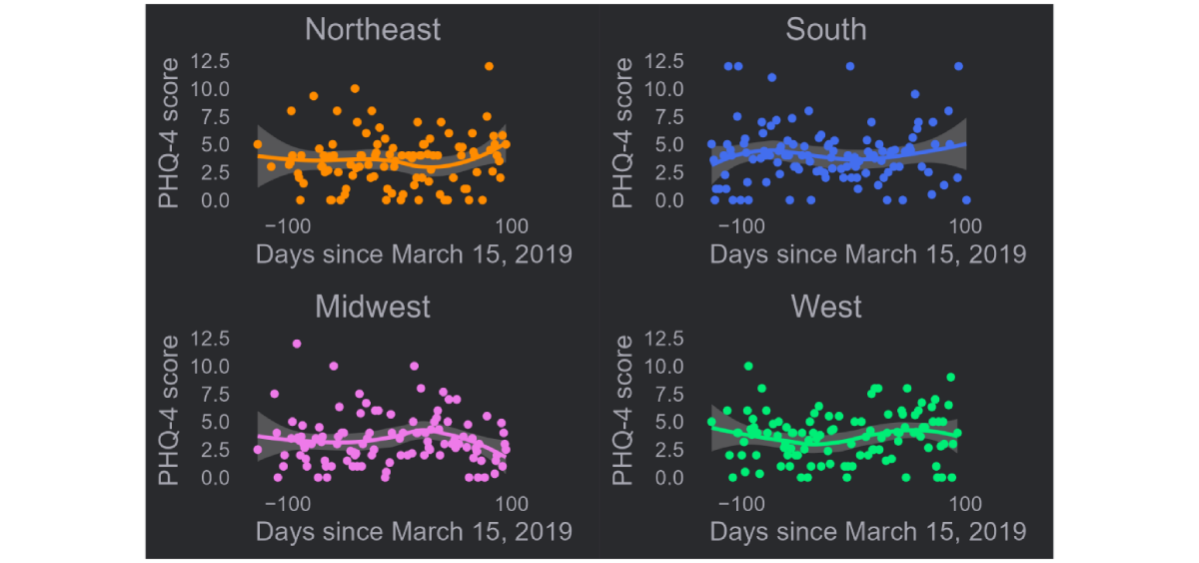
US census region–stratified trends in PHQ-4 scores over time before and 1 year prior to the onset of the COVID-19 pandemic of a prospective US national cohort study of primarily cisgender gay and bisexual men in 2019. PHQ-4: Patient Health Questionnaire-4.

To establish whether a similar trend would be observed in other yearly assessments, the same analysis was conducted for the 12-month assessment (Figure 2). Inspection of the figure reveals a decrease in scores for participants in the Northeast after March 15, 2019, followed by a sharp upturn at the end. Midwest participant scores decreased markedly shortly after March 15, 2019. A small, nonsignificant oscillation in scores was observed among Western participants. No clear trend was observed in scores among Southern participants. All tests for monotonic trends were nonsignificant for 2019 (all *P*>.05; Table 2), indicating, compared to 2020, there was no observed trend in PHQ-4 scores over time.

**Table 1:** Demographics of a prospective US national cohort study of primarily cisgender gay and bisexual men from 2019 to 2020 stratified by US census region with PHQ-4^a^ (anxiety and depressive symptoms) scores.

Demographics		Northeast (n=784)	Midwest (n=780)	South (n=2285)	West (n=1158)	Total (N=5007)	PHQ-4 score, mean (SD)
**Race or ethnicity, n (%)**							
	Black	77 (9.8)	55 (7.1)	330 (14.4)	45 (3.9)	507 (10.1)	2.8 (3.3)
	Hispanic	158 (20.2)	99 (12.7)	580 (25.4)	376 (32.5)	1213 (24.2)	3.1 (3.4)
	White	419 (53.4)	544 (69.7)	1146 (50.2)	568 (49.1)	2677 (53.5)	3.7 (3.5)
	Others or multiracial	130 (16.6)	82 (10.5)	229 (10)	169 (14.6)	610 (12.2)	3.5 (3.6)
**Gender, n (%)**							
	Cisgender men	758 (96.7)	762 (97.7)	2234 (97.8)	1131 (97.7)	4885 (97.6)	3.4 (3.5)
	Transgender men	9 (1.1)	5 (0.6)	18 (0.8)	7 (0.6)	39 (0.8)	5.7 (4.1)
	Transgender women	1 (0.1)	3 (0.4)	10 (0.4)	7 (0.6)	21 (0.4)	1.8 (2.0)
	Others or nonbinary	16 (2)	10 (1.3)	23 (1)	13 (1.1)	62 (1.2)	5.4 (4.1)
**Sexual orientation, n (%)**							
	Gay, queer, or homosexual	697 (88.9)	678 (86.9)	1926 (84.3)	990 (85.5)	4291 (85.7)	3.5 (3.5)
	Bisexual	81 (10.3)	96 (12.3)	328 (14.4)	155 (13.4)	660 (13.2)	3.3 (3.5)
	Straight or heterosexual	0 (0)	2 (0.3)	7 (0.3)	4 (0.3)	13 (0.3)	3.5 (4.1)
	Others	6 (0.8)	4 (0.5)	24 (1.1)	9 (0.8)	43 (0.9)	4.2 (3.9)
Age (years), mean (SD)		32.6 (7.5)	33.5 (8.0)	32.5 (8.1)	33.5 (7.1)	32.9 (7.9)	N/A^b^

^a^PHQ-4: Patient Health Questionnaire-4.

^b^N/A: not applicable.

**Table 2:** The sieve-bootstrap Mann-Kendall test for monotonic trend of Patient Health Questionnaire-4 (depression and anxiety symptoms) of a prospective US national cohort study of primarily cisgender gay and bisexual men from 2019 to 2020. Tests were conducted for each regional time series for each year. Positive τ indicates an increasing monotonic trend.

Region	2019		2020	
	τ	*P* value	τ	*P* value
Northeast	0.05	.44	0.12	.03
Midwest	0.00	.96	0.14	.01
South	0.01	.83	0.00	.96
West	0.07	.26	0.03	.50

## Discussion

### Principal Findings

The region-stratified time series analysis of symptoms of depression and anxiety in the days leading up to and following the onset of the COVID-19 pandemic in the United States revealed significant positive trends among participants in the Northeast and Midwest. These trends were not observed in the South or West, nor were they present the previous year.

The disparate impact of the COVID-19 pandemic on the Northeast [25] as well as heterogeneous COVID-19 policies across the United States may explain the observed trends. Other studies have found that lockdown policies, along with adherence to them, may have resulted in increased depressive symptoms [26].

### Comparison to Prior Work

The findings of significant positive trends in symptoms of depression and anxiety during the onset of the COVID-19 pandemic were consistent with similar studies that found an increased prevalence of depression and anxiety among the general population as well as LGBTQ people during the COVID-19 pandemic [2,6]. However, to our knowledge, this is the first time-series analysis of symptoms of anxiety and depression conducted in an LGBTQ population with data before and after the onset of the COVID-19 pandemic in the United States as well as the first study to identify a regional trend in this population.

Other time series studies have shown the impact of the COVID-19 pandemic on related mental health constructs. A UK study found a significant decrease in referrals to mental health services during the start of the pandemic, although this study was not focused specifically on sexual minorities [14]. Another study of the general population found a decrease in emergency department visits for psychiatric issues [27]. The decrease in mental health service use combined with increased symptoms could indicate a clear, unmet need for mental health care during the beginning of the COVID-19 pandemic. It is important to consider that the pandemic may have had differential effects on sexual minority individuals based on stigma and vulnerabilities. For instance, young adults who had to return to their parents’ homes as a result of the pandemic were found to have faced greater psychological distress [28]. Future studies could explore whether the observed regional effects were modified by changes in living arrangement. Another potential effect modifier could be social support that other studies have found as an important protective effect for sexual minority populations against depressive symptoms [29].

### Limitations

There are some limitations to this study. Survey completion dates were used in calculating days relative to March 15, 2020, and March 15, 2019. It is possible for participants to have started the survey, completed the PHQ-4, and then not completed the survey in its entirety until several days later; however, we do not believe this would have tremendously impacted responses in a meaningful way nor were there enough participants who started before March 15, 2020, and completed it after March 15, 2020, to have had a discernible impact on aggregate results. There also may be other important factors that affect the mental health of sexual minority populations that were not included in this study such as internalized homonegativity [30], social connectedness [31], and minority stress [32]. Finally, the study population was primarily cisgender gay and bisexual men and therefore does not represent the full spectrum of LGBTQ individuals.

### Conclusions

We found evidence of increased depression and anxiety symptoms among a sample of primary cisgender gay and bisexual men during the initial onset of the COVID-19 pandemic in 2020 in the Midwest and Northeast regions of the United States. This trend was not similar among the same study population the previous year nor among participants in the South or West regions of the United States.

## Abbreviations

LGBTQ: lesbian, gay, bisexual, transgender, and queer
PHQ-4: Patient Health Questionnaire-4

## Data Availability

The data sets generated and analyzed during this study are available from the corresponding author on reasonable request.

## References

[1] Mervosh S, Lu D, Swales V (2020). See which states and cities have told residents to stay at home. The New York Times.

[2] Ettman CK, Abdalla SM, Cohen GH, Sampson L, Vivier PM, Galea S (2020). Prevalence of depression symptoms in US adults before and during the COVID-19 pandemic. JAMA Netw Open.

[3] Twenge JM, Joiner TE (2020). Mental distress among U.S. adults during the COVID-19 pandemic. J Clin Psychol.

[4] Riehm KE, Holingue C, Smail EJ, Kapteyn A, Bennett D, Thrul J, Kreuter F, McGinty EE, Kalb LG, Veldhuis CB, Johnson RM, Fallin MD, Stuart EA (2021). Trajectories of mental distress among U.S. adults during the COVID-19 pandemic. Ann Behav Med.

[5] Daly M, Robinson E (2021). Anxiety reported by US adults in 2019 and during the 2020 COVID-19 pandemic: population-based evidence from two nationally representative samples. J Affect Disord.

[6] Gonzales G, de Mola EL, Gavulic KA, McKay T, Purcell C (2020). Mental health needs among lesbian, gay, bisexual, and transgender college students during the COVID-19 pandemic. J Adolesc Health.

[7] Gattamorta KA, Salerno JP, Islam JY, Vidot DC (2021). Mental health among LGBTQ cannabis users during the COVID-19 pandemic: analysis of the COVID-19 cannabis health study. Psychol Sex Orientat Gend Divers.

[8] Flentje A, Obedin-Maliver J, Lubensky ME, Dastur Z, Neilands T, Lunn MR (2020). Depression and anxiety changes among sexual and gender minority people coinciding with onset of COVID-19 pandemic. J Gen Intern Med.

[9] Gato J, Barrientos J, Tasker F, Miscioscia M, Cerqueira-Santos E, Malmquist A, Seabra D, Leal D, Houghton M, Poli M, Gubello A, de Miranda Ramos M, Guzmán M, Urzúa A, Ulloa F, Wurm M (2021). Psychosocial effects of the COVID-19 pandemic and mental health among LGBTQ+ young adults: a cross-cultural comparison across six nations. J Homosex.

[10] Parchem B, Wheeler A, Talaski A, Molock SD (2021). Comparison of anxiety and depression rates among LGBTQ college students before and during the COVID-19 pandemic. J Am Coll Health.

[11] Herrmann WJ, Oeser P, Buspavanich P, Lech S, Berger M, Gellert P (2023). Loneliness and depressive symptoms differ by sexual orientation and gender identity during physical distancing measures in response to COVID-19 pandemic in Germany. Appl Psychol Health Well Being.

[12] Lozano-Verduzco I, Vega-Cauich J, Mendoza-Pérez JC, Craig SL (2023). Perceived social support and mental health indicators of a Mexican LGBT sample during the COVID-19 pandemic. Int J Ment Health Addict.

[13] Krueger EA, Westmoreland DA, Choi SK, Harper GW, Lightfoot M, Hammack PL, Meyer IH (2021). Mental health among Black and Latinx sexual minority adults leading up to and following the 2016 U.S. presidential election: results from a natural experiment. LGBT Health.

[14] Chen S, She R, Qin P, Kershenbaum A, Fernandez-Egea E, Nelder JR, Ma C, Lewis J, Wang C, Cardinal RN (2020). The medium-term impact of COVID-19 lockdown on referrals to secondary care mental health services: a controlled interrupted time series study. Front Psychiatry.

[15] Bauer-Staeb C, Davis A, Smith T, Wilsher W, Betts D, Eldridge C, Griffith E, Faraway J, Button KS (2021). The early impact of COVID-19 on primary care psychological therapy services: a descriptive time series of electronic healthcare records. EClinicalMedicine.

[16] Leske S, Kõlves K, Crompton D, Arensman E, de Leo D (2021). Real-time suicide mortality data from police reports in Queensland, Australia, during the COVID-19 pandemic: an interrupted time-series analysis. Lancet Psychiatry.

[17] Grov C, Westmoreland DA, Carneiro PB, Stief M, MacCrate C, Mirzayi C, Pantalone DW, Patel VV, Nash D (2019). Recruiting vulnerable populations to participate in HIV prevention research: findings from the Together 5000 cohort study. Ann Epidemiol.

[18] Thometz K (2020). Gov. Pritzker orders closure of all Illinois schools, including CPS, over coronavirus concerns. WTTW News.

[19] Pereira S (2020). Breaking: De Blasio announces NYC schools will close through at least April 20th. Gothamist.

[20] Camera L (2020). Seattle public schools close due to coronavirus. US News & World Report.

[21] Census regions and divisions of the United States. U.S. Department of Commerce Economics and Statistics Administration.

[22] Löwe B, Wahl I, Rose M, Spitzer C, Glaesmer H, Wingenfeld K, Schneider A, Brähler E (2010). A 4-item measure of depression and anxiety: validation and standardization of the Patient Health Questionnaire-4 (PHQ-4) in the general population. J Affect Disord.

[23] Wickham H (2016). ggplot2: Elegant Graphics for Data Analysis.

[24] Lyubchich V, Gel YR, Brenning A, Chu C, Huang X, Islambekov U, Niamkova P, Ofori-Boateng D, Schaeffer ED, Vishwakarma S, Wang X (2021). Package 'funtimes'. Functions for Time Series Analysis V 80.

[25] Oster AM, Kang GJ, Cha AE, Beresovsky V, Rose CE, Rainisch G, Porter L, Valverde EE, Peterson EB, Driscoll AK, Norris T, Wilson N, Ritchey M, Walke HT, Rose DA, Oussayef NL, Parise ME, Moore ZS, Fleischauer AT, Honein MA, Dirlikov E, Villanueva J (2020). Trends in number and distribution of COVID-19 hotspot counties—United States, March 8-July 15, 2020. MMWR Morb Mortal Wkly Rep.

[26] Sommerlad A, Marston L, Huntley J, Livingston G, Lewis G, Steptoe A, Fancourt D (2021). Social relationships and depression during the COVID-19 lockdown: longitudinal analysis of the COVID-19 social study. Psychol Med.

[27] Hernández-Calle D, Martínez-Alés G, Mediavilla R, Aguirre P, Rodríguez-Vega B, Bravo-Ortiz MF (2020). Trends in psychiatric emergency department visits due to suicidal ideation and suicide attempts during the COVID-19 pandemic in Madrid, Spain. J Clin Psychiatry.

[28] Salerno JP, Doan L, Sayer LC, Drotning KJ, Rinderknecht RG, Fish JN (2023). Changes in mental health and well-being are associated with living arrangements with parents during COVID-19 among sexual minority young persons in the U.S. Psychol Sex Orientat Gend Divers.

[29] Jacmin-Park S, Rossi M, Dumont L, Lupien SJ, Juster RP (2022). Mental health and social support of sexual and gender diverse people from Québec, Canada during the COVID-19 crisis. LGBT Health.

[30] Davidson K, McLaren S, Jenkins M, Corboy D, Gibbs PM, Molloy M (2017). Internalized homonegativity, sense of belonging, and depressive symptoms among Australian gay men. J Homosex.

[31] Tüzün Z, Başar K, Akgül S (2022). Social connectedness matters: depression and anxiety in transgender youth during the COVID-19 pandemic. J Sex Med.

[32] Fulginiti A, Rhoades H, Mamey MR, Klemmer C, Srivastava A, Weskamp G, Goldbach JT (2021). Sexual minority stress, mental health symptoms, and suicidality among LGBTQ youth accessing crisis services. J Youth Adolesc.

